# Stable and High-Performance Polyaniline/V_2_CT_x_ MXene Composite Electrochromic Films Prepared by One-Pot Electrodeposition Method

**DOI:** 10.3390/polym17223079

**Published:** 2025-11-20

**Authors:** Dan Zhou, Qihuang Deng, Liping Yang

**Affiliations:** 1 Chongqing Key Laboratory of Extraordinary Bond Engineering and Advanced Materials Technology, College of Materials Science and Engineering, Yangtze Normal University, Chongqing 408100, China; 2 Research Department, Wankai New Materials Co., Ltd., Haining 314415, China

**Keywords:** electrochromic property, polyaniline, V_2_CT_x_, MXene, composite film, electrodeposition method

## Abstract

In order to improve the electrochromic performance of polyaniline (PANI), porous PANI/vanadium carbide MXene (PANI/V_2_CT_x_) composite electrochromic films were prepared via a rapid, facile, and low-cost one-pot electrodeposition method from an aqueous solution composed of aniline and V_2_CT_x_ for the first time. The addition of V_2_CT_x_ with a 2D layered structure results in the PANI/V_2_CT_x_ composite films exhibiting significantly different morphologies, structures, electrochemical and electrochromic properties from the pure PANI film. The results show that compared with the pure PANI film, the composite film with optimum V_2_CT_x_ content possesses superior electrochromic properties, such as higher optical contrast, switching speed, coloration efficiency, and cycling stability. The improved electrochromic properties of the composite film can be ascribed to its unique porous morphology and strong hydrogen bond and/or electrostatic interaction between PANI and V_2_CT_x_. This research demonstrates that the one-pot electrodeposition method and the prepared conductive PANI/MXene composite films have potential applications in various fields.

## 1. Introduction

In recent years, with the rapid development of our society, energy crises and environmental pollution have become increasingly serious. Energy conservation and emission reduction have become the consensus of all mankind. In this context, green and energy-saving electrochromic (EC) materials have aroused great interest, which can reversibly change their light absorption/reflection properties under applied voltages and thus can be applied in smart windows to dynamically adjust the indoor temperature and natural lighting of the building [1]. Apart from smart windows, EC materials also show great potential in various devices such as energy-efficient E-paper, information displays, adaptive camouflage apparatuses, anti-glare rearview mirrors, coloration-changing sunglasses, and light shutters [1,2]. EC materials play a pivotal role in the development of the next generation’s energy-saving and environmentally friendly technologies.

Conductive polymers and transition metal oxides are widely studied EC materials [3,4,5,6,7,8]. Among them, polyaniline (PANI) is considered to be one of the most suitable candidates due to its multicolor, high optical contrast, fast switching speed, low cost, and easy preparation by facile methods [9,10]. However, the EC performance parameters of PANI have not yet fully met the practical requirements, especially in terms of poor electrochemical stability during the redox processes, which hinder its widespread application [11,12,13].

A great deal of work has been carried out to improve the EC performance of PANI over the years. Compositing PANI with inorganic nanomaterials is one of the most efficient strategies. For instance, nanomaterials such as TiO_2_ [14], WO_3_ [15], MnO_2_ [10], carbon nanotubes [16], graphene [17], and carbon quantum dots [18] were utilized to fabricate PANI-based composite materials, which showed improved EC performances compared with pure PANI. Composite materials consisting of inorganic and organic components can possess unique advantages for broad applications by the synergistic effect of their constituent components [18]. However, the preparations of the above-mentioned PANI composite films usually require harsh conditions and a tedious synthesis process, such as two or more steps of chemical reactions to separately prepare polymer and nanomaterials, which are time-consuming and not very cost-effective. Therefore, the construction of composite films using simple and effective methods remains scientifically challenging.

Among various techniques for the preparation of EC films, in situ electrodeposition is one of the most promising approaches, particularly for depositing thin and uniform films directly on a large-area substrate of complex shape with good adhesion and high reproducibility under mild conditions in one step [18,19,20]. The thickness, growth rate, micro- and nano-structures of the thin films can also be modulated by simply adjusting applied current, potential, bath chemistry, and temperature [21,22].

Recently, MXenes, a new family of two-dimensional (2D) transition metal carbides or nitrides with layered structure, have emerged and gained increasing interest [23,24] because of unique layered morphology, high electrical conductivity, remarkable electrochemical activity, hydrophilicity, and mechanical properties. The chemical formula of MXenes can be expressed as M_n+1_X_n_T_x_, where M is an early transition metal (e.g., Ti, V, Zr, Nb, Mo, Cr, etc.), X is C and/or N, T_x_ represents surface functional groups (–F, –OH, –O, etc.), and n can take the values of 1, 2, 3 or 4 [25]. These unique characteristics render MXenes attractive candidates for versatile applications in energy storage, transparent conductors, catalysis, energy harvesting, etc. [26,27,28,29]. Likewise, the remarkable properties also make MXenes attractive materials for EC applications [26,30,31]. The earliest and most extensively studied MXene is titanium carbide (Ti_3_C_2_T_x_). In previous studies, Ti_3_C_2_T_x_ was combined with WO_3_, TiO_2_, PANI, or Poly (3,4-ethylenedioxythiophene) (PEDOT) to obtain composite EC materials [27,30,32,33,34,35] and could effectively enhance the electronic conductivity and the ion transport rate of the materials. As another important member of the MXene family, vanadium carbide (V_2_CT_x_) has received attention in the past few years because V_2_CT_x_ has comparable chemical properties with Ti_3_C_2_T_x_, lower ion transport barriers, and more oxidized forms of vanadium elements [36] and has not been much explored to date [37], which provides a great opportunity for constructing composite materials based on V_2_CT_x_. Therefore, it can be expected that combining V_2_CT_x_ with PANI can obtain high-performance EC materials and devices with improved interfacial charge transfer, ion transport, and long-term stability. So far, there have been few reports about compositing V_2_CT_x_ with PANI as EC materials, although PANI/V_2_CT_x_ composites have been studied as supercapacitor materials [36].

In this work, a facile one-pot potentiostatically anodic electrodeposition from aniline and V_2_CT_x_ was conducted to prepare PANI/V_2_CT_x_ composite films, and their EC properties were investigated for the first time. This one-step in situ electrodeposition not only makes the preparation process much simpler and more efficient, but also creates an intimate interface between PANI and V_2_CT_x_ with the unique 2D layered structure, high electrical conductivity, and abundant surface functional groups. The effects of incorporating V_2_CT_x_ into PANI on structures, morphology, electrochemical and electrochromic properties of the composite films were studied in detail. The unique morphology and the interactions between PANI and V_2_CT_x_ have significant enhanced the optical contrast, switching speed, coloration efficiency and long-term cycling stability of the resulting composite films.

## 2. Materials and Methods

### 2.1. Materials

Aniline, dodecylbenzene sulphonic acid (DBSA), and Sulfuric acid (H_2_SO_4_) were purchased from Aladdin Chemical Reagent Co., Ltd. (Shanghai, China). Aniline was purified by reduced-pressure distillation before use. V, Al and C powder were obtained from Aidun Spraying Co., Ltd. (Xingtai, China), Tianjin Gaoke New Material Technology Co., Ltd. (Tianjin, China), and Chuangying Metal Materials Co., Ltd. (Xingtai, China), respectively. Concentrated hydrochloric acid (HCl) and lithium fluoride (LiF) were procured from Chongqing Pinyu Chemical Co., Ltd. (Chongqing, China) and Weng Jiang Regent (Shaoguan, China), respectively. Conducting indium tin oxide-coated glass (ITO/glass, <7 ohm/sq) was purchased from Kaivo Optoelectronic Technologies Co. (Zhuhai, China), and cleaned by ultrasonication in a series of solvents, including detergent, deionized H_2_O, acetone and isopropanol for 10 min, respectively, prior to use. Then, a plasma treatment for 240 s was applied to ITO/glass for further cleaning and improving its hydrophilicity.

### 2.2. Preparation of V_2_CT_x_

V_2_CT_x_ MXene was prepared via synthesis of V_2_AlC particles using a hot-pressing process followed by selective etching of the Al atom layers from V_2_AlC particles using HCl and LiF according to the protocol reported previously [38].

### 2.3. Preparation of PANI/V_2_CT_x_ Composite Films

Three samples of V_2_CT_x_ powder with different contents (3, 5, and 10 wt.% relative to aniline) were dispersed in deionized (DI) water by sonication for 20 min, respectively. Then, DBSA (0.2 M) and aniline monomer (0.025 M) were added to the above dispersions and stirred to form three homogeneous electrodeposition electrolytes.

To prepare EC films on ITO/glass, electrodeposition experiments from the electrolyte were carried out using a potentiostaticcally anodic method at a potential of +1.0 V in a three-electrode system (ITO/glass as working electrode, Ag/AgCl as reference electrode, and Pt as counter electrode) with an electrodeposition time of 400 s. After electrodeposition, the resulting films coated on an ITO/glass electrode were rinsed thoroughly with water and dried in a vacuum oven for 12 h. The as-prepared films are denoted as PANI/V_2_CT_x_-3%, PANI/V_2_CT_x_-5%, and PANI/V_2_CT_x_-10%, respectively. As a comparison, pure PANI film was also prepared under similar conditions except in the absence of V_2_CT_x_ in the solution. The thickness of the films is approximately 220 nm.

### 2.4. Characterization of PANI/V_2_CT_x_ Composite Films

X-ray diffraction analyses of the samples were carried out using a Shimadzu XRD-6100 diffractometer (Tokyo, Japan) over a 2-theta of 5–60° with an X-ray wavelength of 1.542 Å (Cu Kα radiation). FTIR spectra from 400 to 4000 cm^−1^ were recorded on a Nicolet is5 FTIR spectrometer (Thermo Fisher Scientific, Waltham, MA, USA) in ATR mode. X-ray photoelectron spectroscopy (XPS) measurements were conducted on a Thermo Scientific K-Alpha spectrometer (MA, USA) with a monochromatized Al-Kα X-ray source (1486.6 eV). The morphology and microstructure of PANI and PANI/V_2_CT_x_ composite films were observed by a field-emission scanning electron microscope (FESEM, ZEISS Gemini SEM 300, Oberkochen, Germany). The film thickness was measured using an ASIQ Surface Profiler (KLA Tencor, Milpitas, CA, USA).

### 2.5. Electrochemical and EC Measurements of PANI/V_2_CT_x_ Composite Films

Electrochemical properties of the films were tested in a three-electrode electrochemical cell with 1 M H_2_SO_4_ aqueous solution as the electrolyte, where Pt sheet, Ag/AgCl, and as-prepared films coated on ITO/glass were used as counter, reference, and working electrodes, respectively. The electrochemical impedance spectra (Nyquist plots) of the films were measured at a perturbation voltage of 10 mV in the frequency range from 10 kHz to 0.01 Hz using an AUTOLAB PGSTAT 302N potentiostat/galvanostat analyzer equipped with a frequency response analyzer module (Metrohm Autolab, Utrecht, The Netherlands). Cyclic voltammetry (CV) plots were collected on AUTOLAB from −0.4 to +0.4 V at a scan rate of 100 mV/s. All current densities were normalized to the geometric surface area of the electrodes.

EC property measurements of the films, including UV-Vis transmittance spectra from 400 to 800 nm and dynamic switching curves at a fixed monochromatic wavelength of 700 nm, were carried out on a Shimadzu UV-3600 spectrophotometer (Tokyo, Japan), by applying constant potentials and square-wave potentials (oscillating between +0.4 V and −0.4 V at a time step of 50 s or 20 s for stability tests) using the AUTOLAB, respectively.

## 3. Results and Discussion

### 3.1. Preparation and Characterization of V_2_CT_x_ and PANI/V_2_CT_x_ Composite Films

**Figure 1:**
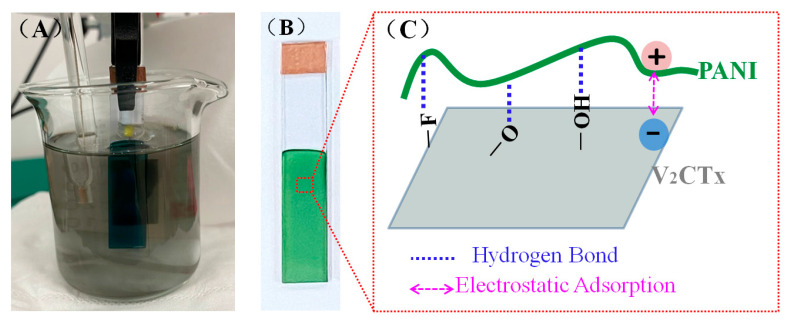
(**A**) Digital photograph of the PANI/V_2_CT_x_ composite film undergoing electrodeposition in electrolyte containing V_2_CT_x_, (**B**) digital photograph of the PANI/V_2_CT_x_ composite film on ITO/glass, and (**C**) schematic illustration of hydrogen bond and/or electrostatic adsorption between V_2_CT_x_ and PANI in the PANI/V_2_CT_x_ composite films.

The end functional groups T_x_ (–O–, –OH, and –F) were introduced into the V_2_CT_x_ surface after the etching process using HCl and LiF, and can contribute to interacting with other polar groups. Because of the existence of a large number of hydrophilic end groups, the prepared V_2_CT_x_ nanosheets were easily and stably dispersed in water or electrodeposition electrolytes through ultrasonic treatment and formed stable solutions, as shown in Figure 1A.

The PANI/V_2_CT_x_ composite films deposited on ITO/glass, as shown in Figure 1B, were prepared by a simple and effective one-step in situ electrodeposition method at +1.0 V from the solutions containing both aniline and V_2_CT_x_. During the electropolymerization of PANI, the organic aniline monomers lose electrons on the working electrode to form cationic radicals, which couple to each other and form polymer chains. Meanwhile, the negatively charged V_2_CT_x_ moves toward the working electrode driven by an electric field, forms the hydrogen bonds and/or electrostatic adsorption interaction with PANI (Figure 1C), and then dopes into the polymer chains to produce a composite film in the cationic radical coupling process [18]. The hydrogen bonds most probably originate from the N-H groups or N of the PANI chains/aniline monomers and terminal groups (–O–, –OH, and –F) on the V_2_CT_x_ surface. The electrostatic adsorption refers to the interaction between the positive charges of protonated PANI/aniline by DBSA and the negative charges of surface terminal groups of V_2_CT_x_. The composite films with PANI as a host penetrated by 2D conductive V_2_CT_x_ could possess not only higher conductivity than the pure PANI film but also unique morphology and structure different from the pure PANI film, greatly improving the electrochemical performance of the PANI film [18].

**Figure 2:**
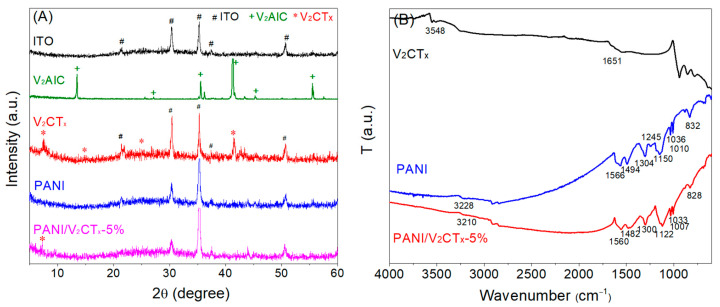
(**A**) X-ray diffraction (XRD) patterns of ITO/glass, V_2_AlC, V_2_CT_x_, PANI film and PANI/V_2_CT_x_-5% film and (**B**) FTIR spectra of V_2_CT_x_, PANI film and PANI/V_2_CT_x_-5% film.

X-ray diffraction (XRD) was employed to investigate the crystal pattern information of the samples. Figure 2A shows the XRD patterns of ITO/glass and the as-prepared V_2_AlC, V_2_CT_x_, PANI and PANI/V_2_CT_x_-5% films on ITO/glass substrates. The XRD pattern of V_2_AlC shows a series of diffraction peaks at 13.5, 27.1, 35.5, 41.3, 45.3 and 55.5° assigned to the crystallographic planes (002), (004), (100), (103), (104) and (106) of the V_2_AlC layered structure, respectively [25], which confirms the successful preparation of V_2_AlC. As shown in the XRD pattern of V_2_CT_x_, in addition to the peaks corresponding to the ITO substrate, the diffraction peaks at 7.5, 14.8, 24.7 and 41.4° are attributed to the crystal indices of (002), (004), (006) and (101) planes of hexagonal V_2_CT_x_ with a multilayered structure [38]. The XRD pattern of PANI film displays no obvious characteristic peak except for a broad and weak hump between 20° and 30°, indicating the amorphous nature of the PANI film [33]. The XRD pattern of PANI/V_2_CT_x_-5% film shows the characteristic diffraction peak at 7.3° corresponding to (002) crystal plane of the pristine V_2_CT_x_, which indicates that V_2_CT_x_ successfully entered into PANI and formed a composite film after electropolymerization of PANI. This peak at 7.3° is relatively weak, and other peaks of V_2_CT_x_ are not prominent, which might be due to the low content of V_2_CT_x_ in the composite film.

To characterize the chemical structure and the intermolecular bonds formed in the composites, FTIR spectra were tested. FTIR spectra of the V_2_CT_x_, PANI and PANI/V_2_CT_x_-5% films are shown in Figure 2B. There are two absorption bands of V_2_CT_x_ at 3548 and 1651 cm^−1^, attributed to the hydroxyl groups on the surface and interlayer or adsorbed water of V_2_CT_x_ [33]. The vibrations of V–O, V–C and V–F are recorded in the low wavenumber region below 800 cm^−1^ [39]. As shown in the FTIR spectrum of the PANI film, the broad peak located at 3228 cm^−1^ is attributed to the N–H stretching vibration of an aromatic amine. The bands at 1566 and 1494 cm^−1^ are due to the C=C stretching vibration of quinoid and benzenoid rings, respectively [40], demonstrating the formation of PANI in the emeraldine state [41]. The bands at 1304 and 1245 cm^−1^ are due to C–N and C=N stretching vibrations, respectively [42]. The band at 1150 cm^−1^ corresponds to N=Q=N (Q meaning the quinoid ring) vibration, which is described as an electronic-like band and is considered a measurement of electron delocalization of PANI [43,44]. The band at 832 cm^−1^ is associated with the out-of-plane deformation of C–H in the 1,4-disubstituted benzene ring. The DBSA doping to PANI is confirmed by the presence of characteristic bands due to the symmetric and asymmetric O=S=O stretching vibrations of the -SO_3_H group of DBSA (1036 and 1010 cm^−1^) [45]. As for the PANI/V_2_CT_x_-5% composite film, the FTIR spectrum shows all the characteristic absorption bands of PANI and less obvious peaks of V_2_CT_x_ due to its relatively low content. The presence of V_2_CT_x_, however, causes significant changes in the band shape and position of PANI owing to the interaction (hydrogen bond and/or electrostatic adsorption) between PANI and V_2_CT_x_. For example, the band due to N–H stretching vibration shifts to lower wavenumbers, from 3228 cm^−1^ for the pure PANI film to 3210 cm^−1^ for the composite film, because the N–H groups of PANI form hydrogen bonds with the –O/–OH/–F functional groups on the V_2_CT_x_ surface, resulting in a change in the vibrational energy level. It is noteworthy that the bands of quinone and benzene rings (1566 and 1494 cm^−1^) red shift to 1560 and 1482 cm^−1^, respectively, suggesting the enhanced electron delocalization and electrical conductivity in the composite film. Moreover, the N=Q=N vibration band shifts from 1150 cm^−1^ to 1122 cm^−1^ and reveals increased intensity and width, further demonstrating the increased electron delocalization and doping level of PANI [43,44] caused by the addition of V_2_CT_x_ and the resultant interaction between PANI and V_2_CT_x_.

**Figure 3:**
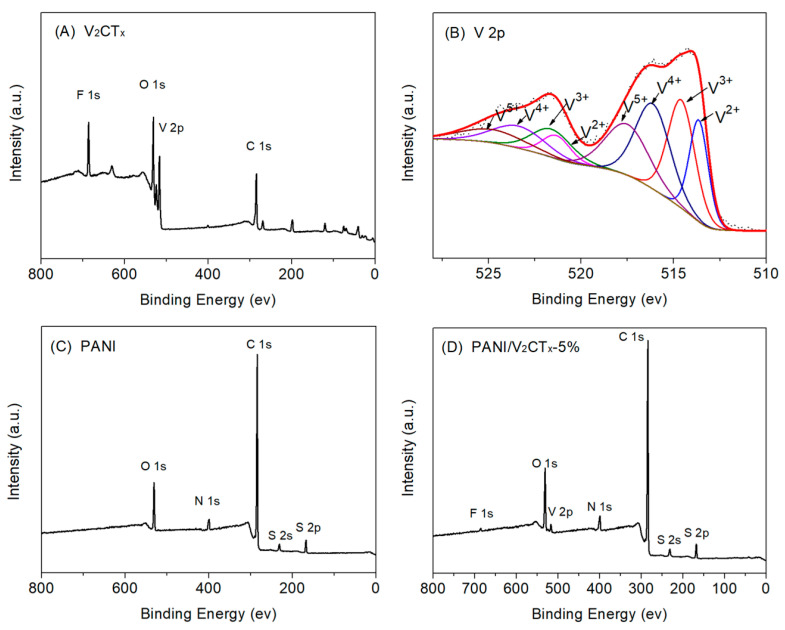
(**A**) XPS wide-scan of V_2_CT_x_, (**B**) V 2p core-level spectrum of V_2_CT_x_, (**C**) XPS wide-scan of the PANI film and (**D**) XPS wide-scan of the PANI/V_2_CT_x_-5% film.

X-ray photoelectron spectroscopy (XPS) analysis was used to analyze the surface elemental composition and chemical states of the samples. Figure 3 shows the survey spectra of V_2_CT_x_, PANI, and PANI/V_2_CT_x_-5% films and the V 2p high-resolution scan of V_2_CT_x_. Apart from V and C, the XPS spectrum of V_2_CT_x_ (Figure 3A) also shows the presence of F and O, which demonstrates that many F- and O-containing groups exist on the surface of V_2_CT_x_, which can endow V_2_CT_x_ with unique properties, such as hydrophilicity, reactivity, electronegativity, etc. The V 2p high-resolution spectrum (Figure 3B) is composed of a spin–orbit doublet attributed to the V 2p1/2 and V 2p3/2 states. There are four deconvoluted peaks in the range of 510 to 528 eV, which are ascribed to V^2+^ (513.6 eV for V 2p3/2, 521.3 eV for V 2p1/2), V^3+^ (514.6 eV and 522.0 eV), V^4+^ (516.1 eV and 523.4 eV), and V^5+^ (517.5 eV and 524.8 eV) [46]. Figure 3C displays the existence of C, O, N, and S in the film, indicating the formation of DBSA-doped PANI. The survey spectrum of the PANI/V_2_CT_x_-5% composite film (Figure 3D) indicates the coexistence of PANI and V_2_CT_x_. The above analysis of the XPS spectra can further confirm that the PANI/V_2_CT_x_ composite films were successfully prepared using the electrodeposition method.

**Figure 4:**
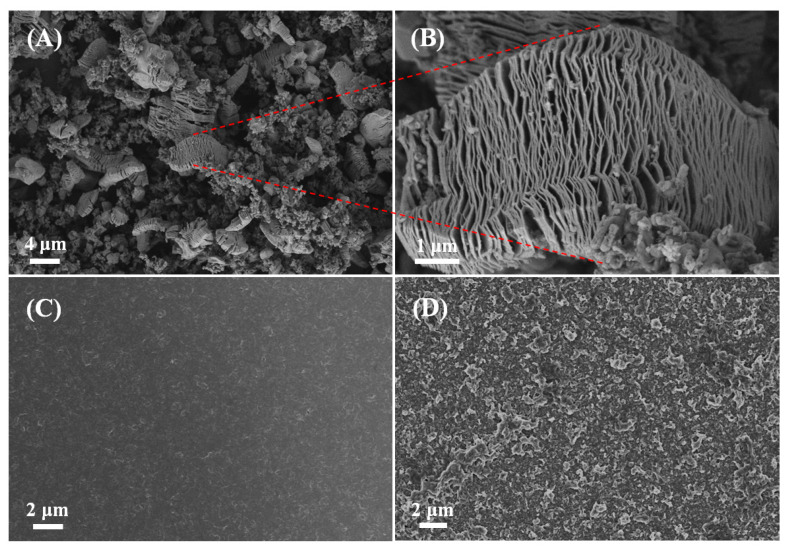
FESEM images of (**A**) and (**B**) V_2_CT_x_, (**C**) the PANI film and (**D**) the PANI/V_2_CT_x_-5% film.

The Scanning electron microscopy was utilized to investigate the structure and morphology of the samples. Figure 4 displays the FESEM images of V_2_CT_x_, the PANI film and the PANI/V_2_CT_x_-5% film. As shown in Figure 4A,B, the V_2_CT_x_ exhibits a characteristic accordion shape, indicating the successful completion of the etching process. According to Figure 4C, the pure PANI film shows a compact and dense morphology. In contrast, the PANI/V_2_CT_x_-5% composite film exhibits a porous and loose surface with the V_2_CT_x_ interleaved into the PANI film uniformly and encapsulated by PANI matrices, as confirmed in Figure 4D. This change in morphology can be attributed to the existence of V_2_CT_x_, avoiding the dense stacking of PANI during electropolymerization via hydrogen bond or electrostatic interaction between PANI and V_2_CT_x_. V_2_CT_x_ with a 2D layered structure and excellent electrical conductivity may contribute to forming an interconnected porous structure and conducting pathways for rapid ionic and electronic transport, a large specific area, and many active sites. Therefore, the unique morphological structure of the composite film will help to improve electrochemical performance [30].

### 3.2. Electrochemical Properties of the Composite Films

**Figure 5:**
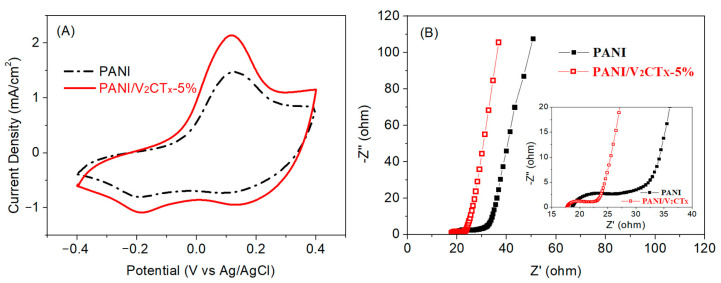
(**A**) CV curves of the PANI and PANI/V_2_CT_x_-5% films at a scan rate of 100 mV s^−1^ and (**B**) Nyquist plots of the PANI and PANI/V_2_CT_x_-5% films at frequency from 10 kHz to 10 mHz using a perturbation amplitude of 10 mV. The inset shows magnified Nyquist plots.

Figure 5A shows cyclic voltammetry (CV) curves of the PANI and PANI/V_2_CT_x_-5% films at a scan rate of 100 mV/s, which possess significant redox peaks due to redox transition between the leucoemeraldine base and emeraldine salt of PANI. Compared to the pure PANI film, the PANI/V_2_CT_x_-5% film displays much higher CV area and current density, which can be attributed to the enhanced redox reaction process caused by a looser and more porous structure and higher conductivity after the incorporation of V_2_CT_x_ into PANI, consistent with the results of structural and morphological studies. The PANI/V_2_CT_x_-5% film with a larger specific surface area, more active sites, and faster ion/electron transport would contribute to the favorable electrochemical performance during the EC process [30].

The charge transfer and ion diffusion behaviors of the films were also demonstrated by electrochemical impedance spectroscopy (EIS). The Nyquist plots of the PANI and PANI/V_2_CT_x_-5% films shown in Figure 5B contain a depressed arc in the high-frequency region and a sloping line in the low-frequency region, indicating that the electrochemical processes are jointly controlled by charge transfer and ion diffusion [33]. The PANI/V_2_CT_x_-5% film shows a smaller intercept of the curve with the Z’-axis, smaller arc diameter, and steeper slope than the PANI film, corresponding to lower equivalent series resistance, lower charge-transfer resistance (Rct) and higher ion diffusion rate for ion insertion/extraction into/from the composite film, respectively [47,48]. This is attributed to the fact that the high conductivity of V_2_CT_x_ and enhanced electron delocalization of PANI/V_2_CT_x_-5% due to the interaction between PANI and V_2_CT_x_, confirmed by FTIR, can help to improve electron transport in the composite film. On the other hand, it may be related to the porous morphology of the PANI/V_2_CT_x_-5% film, and the 2D layered structure of V_2_CT_x_ observed by FESEM study can form more active sites and a shorter ion diffusion path to promote ion diffusion. Therefore, it is expected that the PANI/V_2_CT_x_-5% film possesses improved EC properties, consistent with the CV measurement results.

### 3.3. EC Performance of the Composite Films

**Figure 6:**
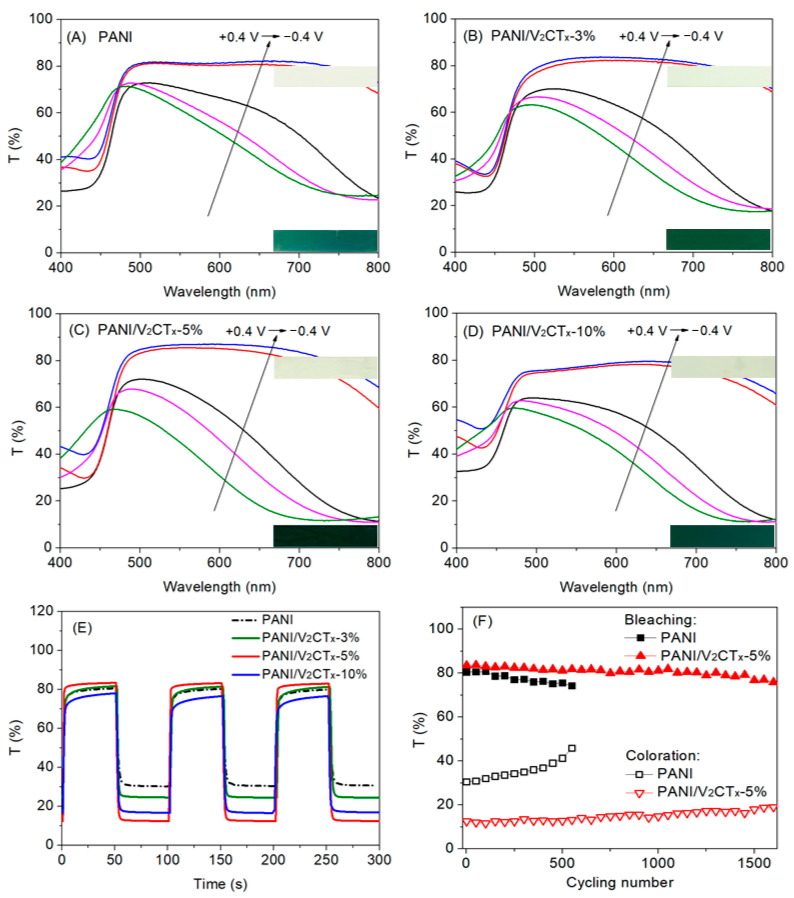
UV-Vis transmittance spectra of (**A**) PANI, (**B**) PANI/V_2_CT_x_-3%, (**C**) PANI/V_2_CT_x_-5% and (**D**) PANI/V_2_CT_x_-10% at different potentials; (**E**) dynamic switching curve comparison between PANI, PANI/V_2_CT_x_-3%, PANI/V_2_CT_x_-5% and PANI/V_2_CT_x_-10% at λ_700nm_ (−0.4 V/+0.4 V, 100 s/cycle); and (**F**) cycling stability of PANI and PANI/V_2_CT_x_-5% (−0.4 V/+0.4 V, 40 s/cycle). Insets in (**A**–**D**) showing the digital photographs of the films electrodeposited on ITO/glass at bleached (upper, light greenish yellow) and colored state (lower, dark bluish green).

Optical contrast, switching time, coloration efficiency, and cycling stability are key parameters for evaluating EC performance of the materials [49]. The effect of V_2_CT_x_ content on EC performance of the PANI/V_2_CT_x_ films was studied, as shown in Figure 6 and Table 1. Compared with the pure PANI film, all composite films exhibit enhanced EC properties, especially the PANI/V_2_CT_x_-5% film with optimum V_2_CT_x_ content.

Optical contrast (∆T) is defined as the difference in the transmittance between the bleached and the colored states at a specific wavelength. According to the UV-vis transmittance spectra in Figure 6A–D, the optical contrasts at λ_700nm_ of the PANI, PANI/V_2_CT_x_-3%, PANI/V_2_CT_x_-5% and PANI/V_2_CT_x_-10% films are 51%, 57%, 71% and 62%, respectively. That is, compositing V_2_CT_x_ with PANI can increase the optical contrast of the PANI film. This is attributed to that compared with the pure PANI film with a relatively compact structure, the composite films possess porous morphology, resulting in more electrochemical active sites, shorter ion diffusion path and easier access of more ions and electrons. This result is consistent with the electrochemical properties as shown in Figure 5. It is worth noting that the PANI/V_2_CT_x_-5% film with optimum V_2_CT_x_ content shows the highest optical contrast (71%) in three composite films, which may be because lower V_2_CT_x_ content (3%) causes slightly porous morphology and higher content (10%) results in restacking or excessive optical absorption of V_2_CT_x_, making less contribution to high EC performance.

The switching times contain coloration and bleaching times, defined as the times for reaching 90% of the maximum transmittance contrast between colored and bleached states. The switching times of the PANI and composite films are obtained from Figure 6E and listed in Table 1. All the films can respond quickly due to the intrinsic property of PANI, in which the PANI/V_2_CT_x_-5% film shows the shortest switching times, i.e., about 1.9 s for bleaching and 2.8 s for coloration. The presence of V_2_CT_x_ with a 2D layered structure makes the PANI/V_2_CT_x_-5% composite film have a more porous morphology, wider ion transport channel, shorter diffusion length and higher conductivity than other films, benefiting faster charge transfer and ion diffusion.

The coloration efficiency (CE) means the optical density change at the wavelength of interest per unit charge inserted into or extracted from the EC films [50]. The CE values of the PANI, PANI/V_2_CT_x_-3%, PANI/V_2_CT_x_-5% and PANI/V_2_CT_x_-10% films are 85, 100, 122 and 107 cm^2^ C^−1^, respectively, as listed in Table 1. The PANI/V_2_CT_x_-5% film exhibits much higher CE than the PANI and the other two composite films, and this is due to more efficient ion diffusion and charge transfer arising from the porous structure. This indicates that small amounts of charge insertion/extraction can result in large optical contrast. This result also guarantees the long-term electrochemical stability of the film.

Cycling stability is crucial to practical applications of the EC materials. Figure 6F displays cycling stability of the PANI and PANI/V_2_CT_x_-5% films, and the optical contrast of the pure PANI film decreases significantly during the cycling test and sustains less than 60% of the initial value after 550 cycles. The pure PANI film shows poor stability because repeated expansion/contraction of the film caused by ion insertion/extraction during the cycling test can damage the structure of the film. Unlike the pure PANI film, the PANI/V_2_CT_x_-5% film possesses much higher stability and still maintains more than 80% of optical contrast up to 1600 cycles, consistent with the results of CE mentioned above. The enhanced stability may be attributed to the unique porous morphology and the strong hydrogen bond/electrostatic interaction between PANI and V_2_CT_x_. The porous morphology can act as a cushion to accommodate the volume change resulting from repeated ion insertion/extraction. At the same time, PANI is strongly bonded with the V_2_CT_x_ sheet through hydrogen bond/electrostatic interaction, which can stabilize the PANI backbones as a reinforcing phase and thus limit the volume change and break-up of the composite film. Moreover, the high conductivity of V_2_CT_x_ facilitates better charge dispersion, thereby avoiding excessive redox reactions in localized areas [30]. As a result, the PANI/V_2_CT_x_-5% film shows superior cycling stability.

## 4. Conclusions

In conclusion, the porous composite films composed of PANI and V_2_CT_x_ have been successfully prepared through a rapid, facile, and low-cost one-pot electrodeposition process. During this process, the presence of V_2_CT_x_ with excellent conductivity and a 2D layered structure contributed to the porous morphology of the composite films. This unique porous morphology and the strong hydrogen bond and/or electrostatic interaction between PANI and V_2_CT_x_ can endow the composite films with many advantages, such as a large specific surface area, many electrochemical active sites, a short ion diffusion path, fast charge transfer and ion diffusion, stabilized PANI backbones, limited volume change, and high conductivity. As a result, the PANI/V_2_CT_x_-5% composite film with the optimum V_2_CT_x_ content exhibits much higher optical contrast, switching speed, coloration efficiency, and cycling stability than the pure PANI film, showing excellent EC properties. This facile and promising electrodeposition method developed in this work opens up a general wet-chemical route to prepare a wide range of low-cost and high-performance PANI-based materials with broad application prospects.

## Figures and Tables

**Table 1:** EC performance of the PANI, PANI/V_2_CT_x_-3%, PANI/V_2_CT_x_-5% and PANI/V_2_CT_x_-10% films at λ_700nm_.

EC Films	∆T (%)	CE (cm^2^ C^−1^)	Bleaching Time (s)	Coloration Time (s)
PANI	51	85	2.7	3.7
PANI/V_2_CT_x_-3%	57	100	2.8	2.9
PANI/V_2_CT_x_-5%	71	122	1.9	2.8
PANI/V_2_CT_x_-10%	62	107	3.3	2.8

## Data Availability

The original contributions presented in this study are included in the article. Further inquiries can be directed to the corresponding authors.
